# Explosion Test and Numerical Simulation of Coated Reinforced Concrete Slab Based on BLAST Mitigation Polyurea Coating Performance

**DOI:** 10.3390/ma15072607

**Published:** 2022-04-01

**Authors:** Ping Lyu, Zhiqiang Fang, Xu Wang, Weibo Huang, Rui Zhang, Yingjie Sang, Pengfei Sun

**Affiliations:** School of Civil Engineering, Qingdao University of Technology, Qingdao 266520, China; fangzhiqiang1015@163.com (Z.F.); wangxu7056@163.com (X.W.); imzray97@outlook.com (R.Z.); yingjiesang@163.com (Y.S.); sun17852720351@163.com (P.S.)

**Keywords:** explosion test, numerical simulation, blast mitigation, polyurea coating, reinforced concrete

## Abstract

The mechanical strength, thermal stability, thermal performance, and microstructure of Qtech T26 blast mitigation polyurea (T26 polyurea) were studied using quasi-static and dynamic mechanical experiments, thermogravimetric experiments, differential scanning calorimetry (DSC), scanning electron microscopy (SEM) experiments, and contact explosion and non-contact explosion experiments with polyurea-coated reinforced concrete slabs. Additionally, the energy dissipation mechanism of the coating was analyzed. The blast mitigation ability and blast mitigation mechanism of T26 polyurea-coated reinforced concrete slabs were investigated by analyzing the macroscopic morphology of reinforced concrete slabs with or without coatings and the contact explosion simulation of polyurea-coated reinforced concrete slabs. The results showed that T26 polyurea exhibited a certain strain rate effect. Its initial thermal decomposition temperature reached 286 °C, and its thermal stability was good. After carbonization, carbon slag can form and adhere to the structural surface. The glass transition temperature Tgs of the soft segment was −44.9 °C, and the glass transition temperature Tgh of the hard segment was 36.5 °C, showing a certain amount of microphase separation morphology. After the explosion test, there was a small pit on the front surface of the coated reinforced concrete plate, and there was no damage on the back surface. The integrity of the plate was good. The uncoated reinforced concrete slab had a large crater on the front of the explosion surface, and the back of the explosion surface experienced explosion collapse, concrete crushing, and an overall loss of stability. The numerical simulation results showed that the failure mode of the coated plate was consistent with the test. The kinetic energy conversion rate of the uncoated reinforced concrete plate was 87.27%, and the kinetic energy conversion rate of the coated reinforced concrete plate was 95.36%. The T26 coating improved the kinetic energy conversion rate of the structure and improved the blast mitigation ability of the reinforced concrete plate structure.

## 1. Introduction

Polyurea is a kind of polymer material formed by the rapid cross-linking of isocyanate components and amino compounds that has excellent mechanical properties [1,2] and which has attracted wide attention from experts and scholars at home and abroad. Subsequently, the explosion protection of polyurea was studied. For the protection of concrete structures, whether it is blasting demolition or anti-terrorism explosion prevention, reducing the damage caused by concrete fragmentation is also a hot topic in current research. Polyurea can be coated on the surface of the structure to improve its explosion resistance. In a study of polymer-reinforced walls by the US Air Force Laboratory, it was found that compared with many other polymer materials, such as carbon fiber and aramid fiber, polyurea showed a better blast mitigation effect in the explosion protection of wall structures [3]. On this basis, James T. Baylot [4] of the U.S. Army Engineering University compared the explosion test of fiber-reinforced polymer, polyurea, and steel plate protective walls. It was found that the wall reinforced by fiber-reinforced polymer and a polyurea coating can maintain better integrity and can coat the wall fragments and debris in the structure to reduce debris splash. Further study showed that the blast mitigation effect when both side walls were polyurea-coated was better than that of a single polyurea-coated side wall [5,6], and for the one-sided coating wall, coating polyurea on the interior face of walls can better cover the wall debris after explosion than the blast-facing side, and with the increase in the thickness of polyurea coating, the blast mitigation effect of the wall was significantly enhanced [7,8].

In addition, the good blast mitigation effect of polyurea in explosion protection is closely related to its excellent mechanical properties and strain rate effect [9]. Through dynamic mechanical tests, Yi [10] and Wang [11] found that polyurea showed a rubber state at a low strain rate, while polyurea showed a leather or glass state at a high strain rate. The stress–strain curves of polyurea were nonlinear and were greatly affected by the strain rate, whether at low or high strain rates. Guo [12] and Qiao [13] had found that polyurea has a good strain rate effect. At a high strain rate, the mechanical properties of the material were significantly improved, which can make it exhibit better explosion resistance under explosion conditions.

Given the limitations of the field explosion test, other relevant research is based on the use of ANSYS/LS-DYNA software for numerical simulation. Amini [14] used numerical simulation to study how polyurea coating can improve the blast resistance of steel plates, which is consistent with the experimental data, confirming the accuracy of the numerical simulation results. Subsequently, simulation studies in various directions were gradually carried out. Chen [15] studied the spallation of polyurea on the back explosion surface and the front explosion surface under explosive load and the kinetic energy change in polyurea fragments using numerical simulation and found that the spalling degree of polyurea was related to the thickness of the coating. The relatively thick coating can absorb more energy and reduce the damage to the structure. Samiee [16] compared the dynamic response of polyurea coating on the front and back blast surfaces under blast load using a simulation and found that the blast mitigation effect of polyurea coating on the back surface of the steel plate was the best. For the coating structure on the back explosion surface, Raman [17] and Ghaderi [18] used a numerical simulation to find that the polyurea coating can improve the displacement control and energy dissipation of reinforced concrete slabs, reduce the strain energy and kinetic energy of the structure, and improve the anti-explosion ability by reducing the displacement and energy absorption and weakening the explosion energy transfer.

Polyurea protective coating has been studied in terms of anti-explosion, but there is little analysis on the influence of the performance of polyurea material on its blast mitigation. The explosion process is complicated. The explosion will produce high temperatures and strong shock waves that are scattered around. In the explosion protection, the coating material should not only withstand the high temperature and high heat, but also resist the tensile and tearing effects caused by the explosion loads. Therefore, the thermal properties and mechanical properties of coating materials are the basic conditions for their blast mitigation effect. In this regard, for blast mitigation, T26 polyurea was selected in this paper. Starting with the characteristics of the material, the basic mechanical properties and thermal properties of T26 polyurea at different strain rates were studied to understand the molecular stability of T26 polyurea at high temperatures and predict the blast mitigation ability of the material. On this basis, through the contact explosion test and non-contact explosion test of T26 polyurea-coated reinforced concrete slabs and the numerical simulation of reinforced concrete slabs with or without polyurea coating under contact explosion, the damage of reinforced concrete slabs with or without polyurea coating was compared, and the energy absorption and energy dissipation characteristics of T26 polyurea under explosion load were analyzed. The microstructure and the failure mode of the cross-section of T26 polyurea coating after the explosion were observed. The failure mode of the coating under explosion load was studied to verify the blast mitigation ability of T26 polyurea.

## 2. Experimental Procedure

### 2.1. Coating and Coated Reinforced Concrete Slab Preparation

#### 2.1.1. Coating Preparation

T26 polyurea is a kind of blast mitigation polyurea material optimized via the traditional polyurea formula. The density of T26 polyurea is below 1 g · cm^−3^ and has the characteristics of low density, as shown in Table 1. T26 polyurea is light and cannot increase the weight of the structure when applied to the protective structure. T26 polyurea was prepared through the reaction of component A isocyanate and component B amino compound at a mass ratio of 1.1:1 and was then sprayed by a PHX-40 host and AP-2 spray gun. After spraying molding and curing at room temperature for 7 days, when the mechanical properties of the material were stable, the mechanical test was carried out.

#### 2.1.2. Preparation of Reinforced Concrete Slab

**Figure 1:**
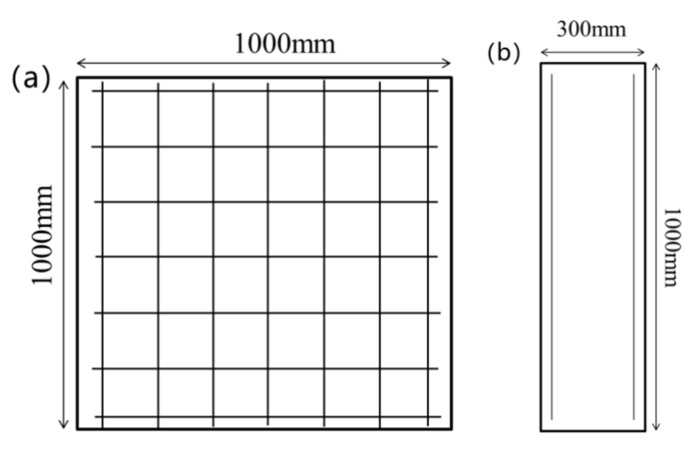
Reinforcement diagram of reinforced concrete slabs: (**a**) front face; (**b**) side face.

The concrete strength grade was C40 with the reinforcement spacing at 150 mm and the diameter at 10 mm, while the protective layer thickness was 15 mm and the size of the reinforced concrete slab was 1000 mm × 1000 mm × 300 mm. The reinforcement was as shown in Figure 1.

#### 2.1.3. Preparation and Experimental Scheme of Coated Reinforced Concrete Slabs

**Figure 2:**
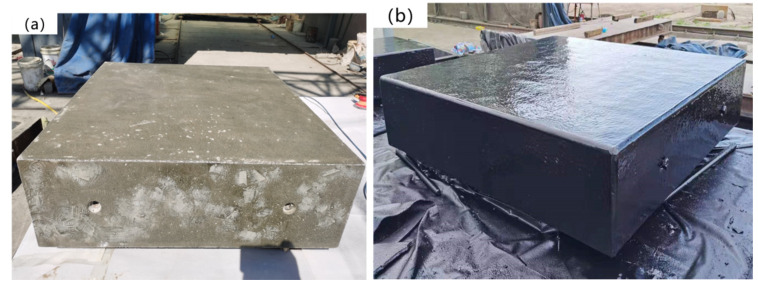
Samples of reinforced concrete slab: (**a**) painted primer; (**b**) sprayed T26 polyurea.

Good adhesion between the T26 coating and the concrete substrate is crucial. Before spraying, an epoxy-modified polyurethane primer should be applied to the surface of the substrate (Figure 2). The sealing ability and bonding ability of the primer are good, and the dust remaining on the substrate surface can be firmly bonded to the substrate surface to reduce the defects, such as pinholes in the substrate. In addition, the penetration ability of the primer is strong, and it can be closely bonded with a concrete and polyurea coating at the same time to enhance the adhesion of the coating. Polyurea was sprayed onto the surface of the reinforced concrete slab using spraying equipment, and the coating thickness was 10 mm, which was achieved with comprehensive spraying. Figure 2 shows the reinforced concrete slab samples sprayed with T26 polyurea and painted primer, and the surface of the samples is smooth and flat.

**Figure 3:**
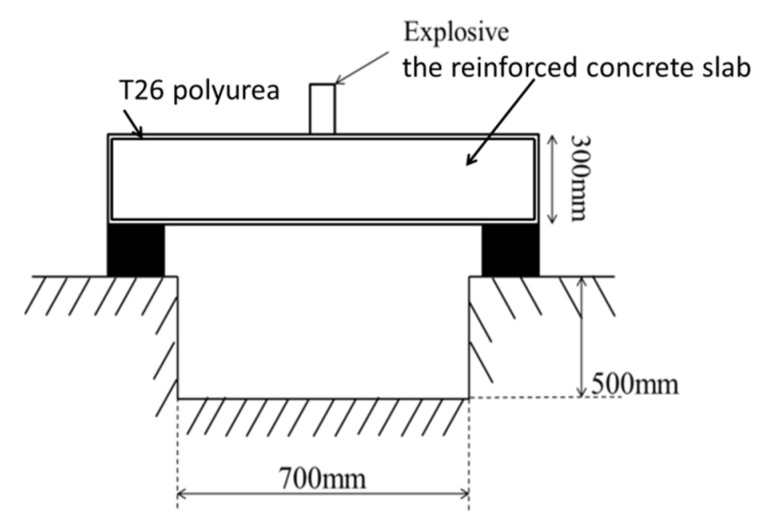
Schematic diagram of explosion experiment setup.

Reinforcement concrete slabs sprayed with T26 were, after seven days of maintenance, placed on the restraint frame. The explosion pit had a side excavation depth of 0.5 m and a width of 0.7 m on the back explosion surface, and explosives with a TNT dosage of 1.4 kg were placed on the upper surface of the reinforced concrete plate for the explosion test. The schematic of the experimental setup is shown in Figure 3. The explosion test was carried out in three groups, and the first group was the control group. The test conditions are shown in Table 2.

### 2.2. Performance Characterization Experiment of Coating

#### 2.2.1. Quasi-Static Tensile Test

**Figure 4:**
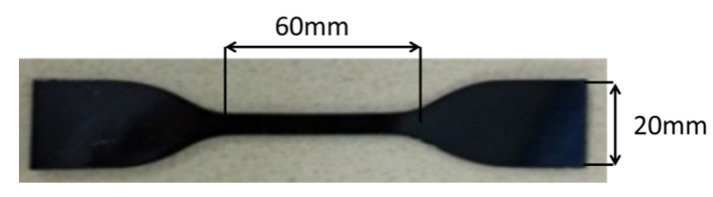
Quasi-static tensile specimens.

The T26 polyurea samples were cut into a dumbbell shape using a tablet punching machine (As shown in Figure 4). The universal testing machine was used to connect the sample and the fixture vertically to ensure the balance of force. The displacement speed was 1.5 mm/min, 15 mm/min, and 150 mm/min, and the corresponding strain rates were 0.001 s^−1^, 0.01 s^−1^, and 0.1 s^−1^. The tensile test was carried out until the samples were broken.

#### 2.2.2. High-Speed Tensile Test

**Figure 5:**
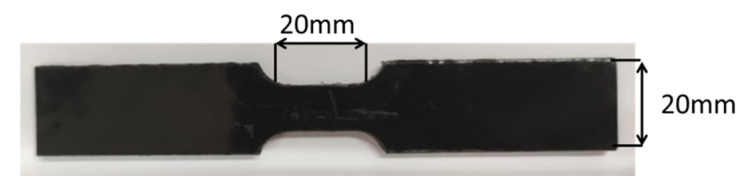
High speed tensile specimens.

The T26 polyurea was cut into the samples shown in Figure 5 using a tablet punching machine. The Instron VHS 160-100/20 high-speed hydraulic servo material testing machine was used for the high-speed tensile test, which required a vertical connection between the samples and the fixture to ensure the balance of force. The displacement changes between fixtures were recorded by the extensometer in the equipment.

#### 2.2.3. Differential Scanning Calorimetry Test

A total of 12.2 mg of T26 sample was weighed and tested using NETZSCH DSC 204 F1 differential scanning calorimeter. To avoid experimental error, this experiment needed to be carried out under conditions with nitrogen. The experimental temperature range was −100~80 °C.

#### 2.2.4. Thermogravimetric Experiment

A total of 5–10 mg of T26 sample was weighed and tested using a differential thermal–gravimetric synchronous thermal analyzer TAQ600. The whole experiment was carried out in a nitrogen environment, and the test time was 3 h, and the temperature range was 3–800 °C.

### 2.3. Explosion Test

#### 2.3.1. Macroscopic Appearance of Reinforced Concrete Slabs

According to the test scheme in Table 3, the damage to the front and back blasting surface of the coated reinforced concrete plate and the uncoated one after an explosion was compared. Setting condition 1 as the control group, the protective effect of T26 coating on the front and back blasting surfaces of reinforced concrete slabs was analyzed.

#### 2.3.2. Microstructure of Coating after the Explosion

The T26 coatings in different explosion regions after the explosion were selected, and the microstructure of the samples was observed using a JSM-7500F scanning electron microscope with a magnification of 4000 times. The resolution of the instrument is 1.0 nm (15 kV) and 1.4 nm (1 kV), the acceleration voltage is 0.1–30 kV, the electron gun uses a tungsten filament lamp, and the magnification is 25–1,000,000 times.

### 2.4. Numerical Simulation

#### 2.4.1. Establishment of Finite Element Models

ANASYS software was used for the modeling, and LS-DYNA software was used to calculate the model. Calculation models included steel bars, concrete slabs, explosives, air, and polyurea materials. A Lagrange grid and hexahedral element were used in the model grid. The fluid–solid coupling algorithm (ALE) was used in the grid, and the non-reflective normal boundary conditions were set at the boundary. The polyurea material was modeled using a shell element, the steel was modeled using a beam element, and the concrete slab was modeled using a solid element. In addition, the coating and concrete automatically had single-sided contact, and the common node model was used between steel and concrete. The reinforced concrete slab model size was 1000 mm × 1000 mm × 300 mm, and the bottom had fixed support using double reinforcement. The explosive was a cylindrical TNT with a radius of 50 mm and height of 110 mm, and the dosage was 1.4 kg. The air was evenly divided into 1.5 cm grids.

After establishing the model of contact explosion between uncoated and T26-coated reinforced concrete slabs in ANSYS, the output file was set as an LS-YDNA file format. The relevant parameters in the output file were modified, including coating thickness and output time, and the coating thickness was 10 mm, the output time was 1000 μs. The modified file was post-processed in LS-YDNA software to obtain the relevant simulation results.

#### 2.4.2. Material Model Selection

**Figure 6:**
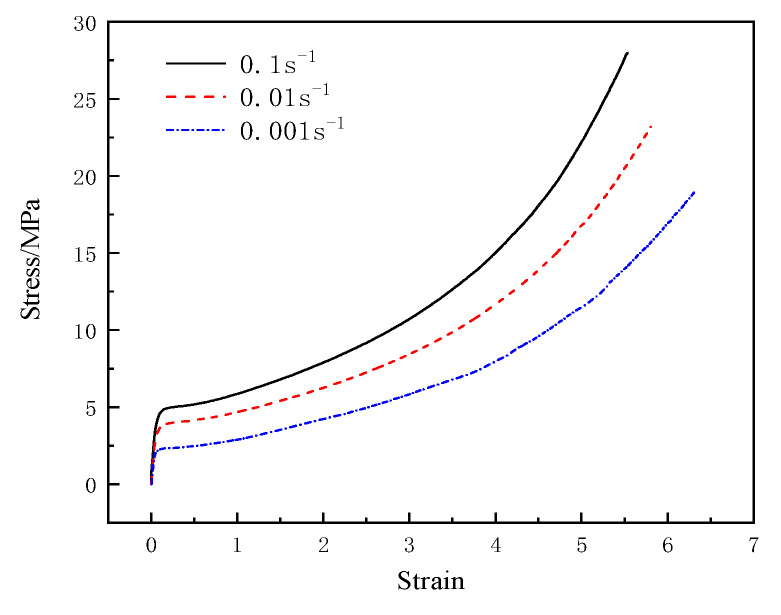
T26 polyurea stress–strain curve at a low strain rate.

**Figure 7:**
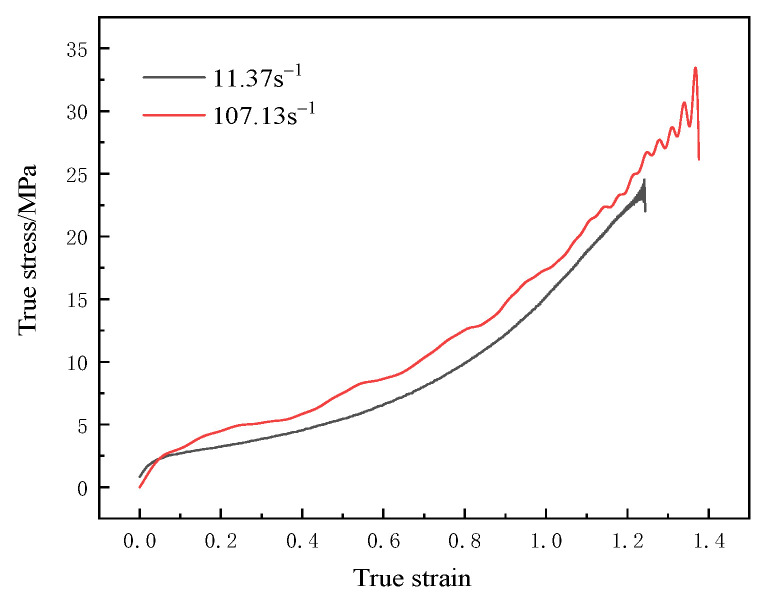
T26 polyurea true stress-strain curve at a high strain rate.

For polyurea, the elastic-plastic model named MAT_PIECEWISE_LINEAR_PLASTICITY [19] was used, which can define elastic-plastic material with an arbitrary stress-strain curve and arbitrary strain rate correlation. The Mooney–Rivlin [20] hyperelastic material model was utilized to describe the behavior of polyurea, which is a function of strain energy. This model is based on incremental updates of stress components and assumes linear viscoelasticity at each time step. It evaluates bulk and shear moduli at each step on the basis of state variables. The stress–strain curve parameters of polyurea are shown in Figure 6 and Figure 7. The simulation parameters of polyurea materials are as shown in Table 4.

Johnson–Holmquist–Cook model [21] was used for concrete materials, and it considers the effects of strain rate, hydrostatic pressure, and damage accumulation on strength. The Johnson–Holmquist–Cook concrete model parameter cards are as shown in Table 5.

The reinforcement model was MAT_PLASTIC_KINEMATIC [22]. It is an elastic-plastic material model with strain rate dependence and failure. The strain rate effect was described by the Cowper-Symonds model. The reinforcement simulation parameters are as shown in Table 6.

The air was described by the MAT_NULL material model and linear polynomial state equation EOS_LINEAR_POLYNOMIAL. The air model parameters are as shown in Table 7 and Table 8.

The explosive model was MAT_HIGH_EXPLOSIVE_BURN, and the EOS_JWL equation of state was selected. The detailed parameters of the above material model can be found in the LS-DYNA user manual [22]. The explosive model parameters are as shown in Table 9 and Table 10.

## 3. Results and Analysis

### 3.1. Mechanical Properties of T26 Coating

#### 3.1.1. Low Strain Rate

Table 1 shows the quasi-static tensile test of T26 polyurea and its measured basic properties.

The stress–strain curves of T26 polyurea at a low strain rate are shown in Figure 6. The figure indicates that at a low strain rate, the stress-strain curves of the material can be roughly divided into three stages: elastic stage, highly elastic stage, and nonlinear deformation stage. When the strain rate increased from 0.001 s^−1^ to 0.1 s^−1^, the stress of the material increased with the increase in the strain rate. Thus, T26 polyurea showed a good strain rate effect at a low strain rate.

#### 3.1.2. High Strain Rate

The strain rate effect of the material under a high strain rate was further studied. The true stress-strain curve of the material under a high strain rate was obtained by filtering the data collected by the acquisition instrument, as shown in Figure 7. Under the same strain, the stress at the strain rate of 107.13 s^−1^ was higher than that at the strain rate of 11.37 s^−1^. The accurate stress–strain curves of T26 polyurea were nonlinear at a high strain rate. With the increase in true strain, the true stress increased, and when the strain rate increased, the true stress also increased, indicating that T26 polyurea presented a good strain rate effect. T26 polyurea showed good mechanical strength at a high strain rate. Therefore, during the explosion, the material can continuously improve its strength to resist explosion shock load and enhance the blast mitigation ability of the composite structure with the increase in explosion load strength.

### 3.2. Thermal Properties of Coatings

#### 3.2.1. Differential Scanning Calorimetry Analysis

**Figure 8:**
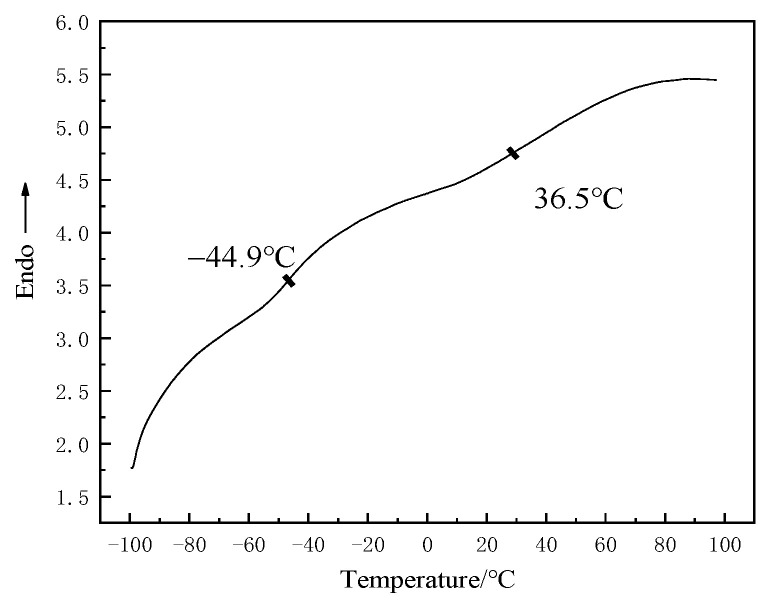
Glass transition temperature of T26 polyurea.

Figure 8 shows the glass transition temperature of T26 polyurea from 100 °C to 80 °C. As can be seen from the diagram, the second phase transition of the material corresponds to its soft glass transition temperature (Tgs), which was −44.9 °C, and the main phase transition corresponds to its hard glass transition temperature (Tgh), which was 36.5 °C. This showed that the T26 polyurea molecular chain is a certain microphase separation structure. In the temperature range from −100 °C to 80 °C, the T26 polyurea material did not appear to have obvious endothermic and exothermic peaks.

**Figure 9:**
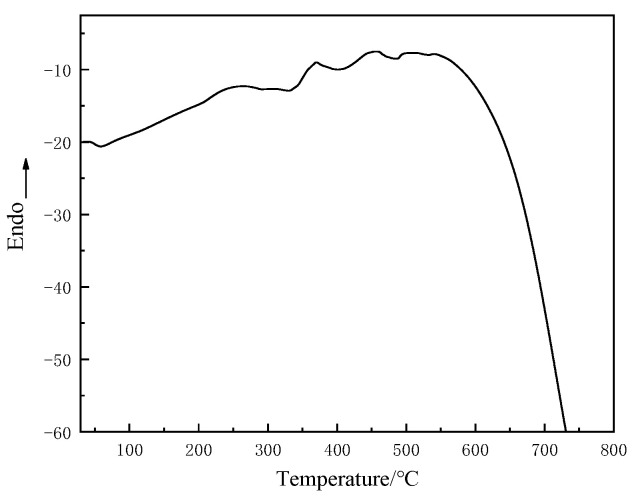
DSC curve of T26 polyurea in a wide temperature range up to high temperatures.

The DSC curve of materials in the temperature range from 30 °C to 700 °C is shown in Figure 9. With the increase in temperature, the crystal melting endothermic peak appeared to be near 375 °C, and there was micro-crystallization in the molecules of the T26 material, as shown in Figure 9. Soft segments in molecular chains determine the flexibility and hard segments determine the strength of materials. Explosives release energy instantly and produce high temperatures and high heat during the explosion. The crystallization and melting of T26 polyurea at a high temperature mean that it can absorb and release energy through crystallization and the melting of the coating. At the same time, in the high-temperature environment, the free volume of the material began to expand gradually, and the molecular chain segment became active and stretched gradually, making the material more flexible. The coating ability of the back blast surface of the structure was stronger, and the anti-fragmentation effect was better. Through the above data, we can observe the material can absorb energy to change its morphology to resist high-temperature damage under a high-temperature environment, which provided a research basis for subsequent explosion tests.

#### 3.2.2. Thermogravimetric Experiments Analysis

**Figure 10:**
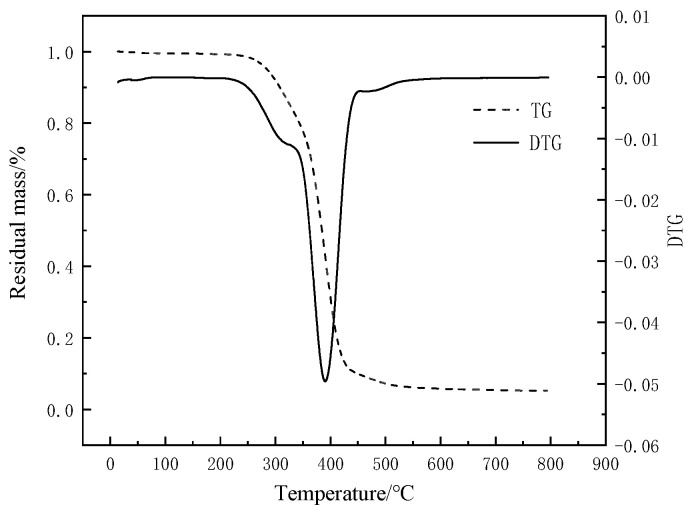
T26 polyurea TG and DTG curves.

The explosion environment is a high-temperature environment, which requires the high-temperature adaptability of materials. The TG and DTG curves of T26 polyurea coating are shown in Figure 10. It can be seen that the thermal decomposition of T26 polyurea material was mainly concentrated in the range of 286–425 °C when examining the TG in Figure 10. Before 286 °C, the uncured moisture and impurities in the material degraded slowly. When the temperature reached 286 °C, the material started thermal decomposition. The temperature was the initial thermal decomposition temperature of the material. With the increase in temperature, the degradation degree of the material increased and the remaining mass decreased. When the temperature reached 425 °C, the mass loss of the material reached 90% of the initial mass. In this process, the molecular chain segment of the material moved under the influence of temperature, the molecular cross-linking structure expanded gradually, and the molecular bond broke. After the temperature reached 700 °C, the material was no longer degraded and basically carbonized. In the explosive high-temperature environment, the polyurea material was decomposed into carbon residue. Carbon slag formed a carbonized layer attached to the surface of the structure, which played a role in coating heat insulation while weakening the damage and failure of the internal structure under explosive load, and it, thus, had a certain protective effect on the structure.

In addition, it can be seen from the DTG curve in Figure 10 that the thermal decomposition rate of the material increased first and then decreased gradually. When the temperature reached 333 °C, the thermal decomposition rate increased slightly. From the DSC experiment of the material, it can be seen that T26 polyurea is a microphase separation form, and the hard segment and soft segment are cross-linked. Therefore, the peak was due to the first decomposition of the hard phase in the material resulting in the first phase of the material quality loss. Then, with the increase in temperature, the decomposition rate of the material increased, and the thermal decomposition rate of the material was the largest at 386 °C. This was mainly due to the decomposition of the benzene ring and its soft segment in the material, resulting in the mass loss of the second stage of the material.

### 3.3. Morphology Study after Explosion

#### 3.3.1. Macroscopic Morphology

**Figure 11:**
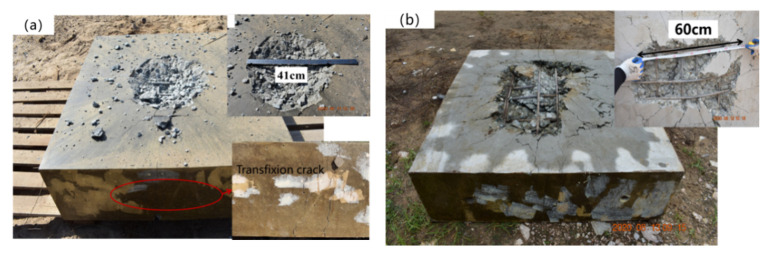
Failure modes of uncoated reinforced concrete slab after contact explosion: (**a**) face surface blastblast; (**b**) back blast surface face surface blast.

The failure modes of uncoated reinforced concrete slabs after contact explosion are shown in Figure 11. It can be seen in Figure 11a that the central area of the uncoated reinforced concrete slab was seriously damaged, the concrete was broken and the steel bar was exposed. A large number of explosive fragments appeared on the surface and around the reinforced concrete slab. At the same time, there was a pit in the center of the reinforced concrete slab and a large number of cracks spread around the edge of the pit. The size of the pit was measured, and the maximum diameter and depth of the failure in the center area were 41 cm and 11.2 cm, respectively. Through Figure 11b, it can be seen that the concrete had been broken and broken off, the steel bars experienced yield failure, and a large number of cracks spread around the failure zone. The maximum diameter of the failure area of the back blast surface was 60 cm, which is significantly larger than that of the blasting surface. Additionally, the destruction debris of back blast surface was more than that facing the blast. Thus, to avoid a large number of fragments splashing, the emphasis should be placed on the back protection of reinforced concrete slabs. Further observation shows that there were a large number of vertical cracks on the side of the uncoated reinforced concrete slabs, and the cracks extended up and down to the surface of the structure (Figure 11a). The overall yield failure of the reinforced concrete slab had occurred, and the bearing capacity was completely lost.

**Figure 12:**
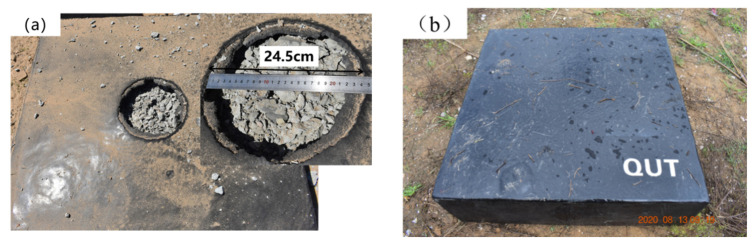
Failure modes of reinforced concrete slab coated with T26 after contact explosion: (**a**) blast face surface blast; (**b**) Face surface blastback blast surface.

The failure modes of the reinforced concrete slabs coated with T26 after contact explosion are shown in Figure 12. There was a pit in the central area of the face surface blast blast of the T26-coated reinforced concrete slab. The coating on the edge of the pit protruded upward, and the failure area was approximately circular. The concrete in the pit was broken, and a small amount of explosive debris was dispersed around the concrete slab, and the debris particles were small. The pit diameter was measured, and the maximum failure diameter of the central area was 24.5 cm, and the maximum depth was 9.4 cm, as shown in Figure 12a. Compared with the uncoated reinforced concrete slab, the maximum failure diameter in the center area was reduced by 40%, and the maximum depth was reduced by 16.07%. The back blast surface of coated reinforced concrete slab was intact, the polyurea coating was not damaged, and the overall structure still had a certain bearing capacity, as shown in Figure 12b. Compared with the explosion on the uncoated reinforced concrete slab, after the contact explosion of the reinforced concrete slab with the T26 coating, the coating had perforation failure, but it did not spread to the surrounding cracks. The overall structure of the reinforced concrete slab was intact, and there was no overall yield failure, but rather local damage. Therefore, the T26 coating can effectively improve the blast resistance of reinforced concrete slabs.

**Figure 13:**
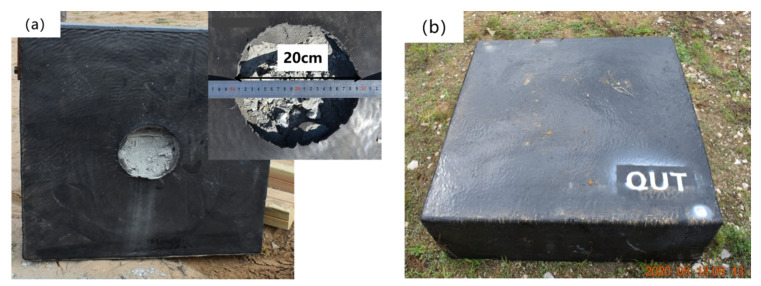
Failure modes of coated reinforced concrete slab after explosion with explosion distance of 50 mm: (**a**) blast face surface blast; (**b**) face surface blastback blast surface.

The failure modes of the reinforced concrete slab coated with T26 after the explosion with an explosion distance of 50 mm are shown in Figure 13. There was a near-circular damage pit in the central area of the face surface blast blast of the coated reinforced concrete slab, and the coating on the edge of the pits slightly rose. There were a small number of concrete fragments in the center of the pit and there were no cracks in the coating. The overall structure of reinforced concrete slabs was intact, but local damage occurred. The maximum failure diameter of the pit was 20 cm, as shown in Figure 13a. The maximum diameter of the reinforced concrete slab pit with and without coating protection under contact explosion was reduced by 18% and 51%, respectively. There was no damage to the back blast surface of the coated reinforced concrete slab, as shown in Figure 13b. Under non-contact explosion, the damage degree of coated reinforced concrete slab was lower, and the protective effect of T26 polyurea was better.

#### 3.3.2. Micromorphology

**Figure 14:**
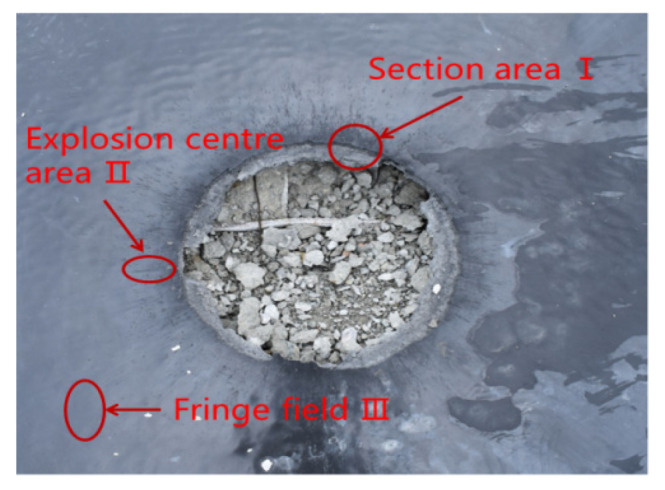
Different explosion regions of coating.

The T26 polyurea coating after explosion damage was divided into section area I, explosion source center area II, and coating edge area III, and the coatings in the three regions were cut for microscopic morphology observation, as shown in Figure 14.

**Figure 15:**
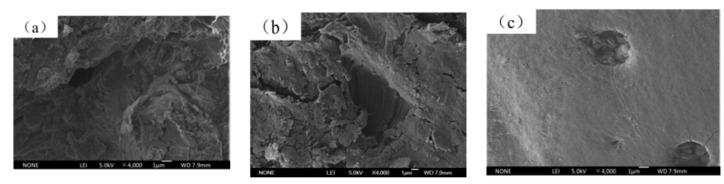
Microstructure of T26 polyurea coating after explosion: (**a**) section area I; (**b**) explosion source center area II; (**c**) coating edge area III.

The micro-morphology of T26 polyurea coating was enlarged by 4000 times in different regions after the explosion. The surface of the section area was torn in different degrees, the shape of the damage was scaly, the surface arrangement was disordered and uneven, and there were more tearing layers and a small amount of brittle failure. It can be seen that the polyurea coating melted in some areas under the high temperature caused by the explosion, and the strong shock wave caused the tensile failure of the polyurea coating. At the end of the explosion, the instantaneous decrease in temperature led to the transformation of polyurea from a highly elastic state to a glass state, and the broken polyurea solidified into flakes attached to the surface of the section, as shown in Figure 15a. The cross-section of the polyurea coating was not uniform, and the microstructure was dense. Large plastic deformation leads to obvious tearing failure, accompanied by more cracks, and melting phenomenon occurs in some areas, as shown in Figure 15b. T26 polyurea was subjected to high temperatures and strong shock waves at the same time when resisting the explosion. The shock wave made the polyurea coating bear different directions of impact force, resulting in irregular tearing damage and a large number of cracks on the surface of polyurea coating. The surface of the coating edge area was relatively smooth, and there was no melting and tearing failure, indicating that the edge coating was less affected by the explosion load, and that most of the energy generated by the explosion was released at the center. However, there were many tiny cracks, and the cracks extended from the concave area to the surrounding, as shown in Figure 15c. The regular shape of the concave area in the figure may be due to the destruction of the bubbles in the raw material during the spraying process, resulting in a round hole on the surface. Under the explosion impact, the strength at the round hole was low, resulting in the crack extending from the round hole to the surrounding area. Therefore, it is particularly important to minimize the generation of bubbles in the spraying process of polyurea.

### 3.4. Numerical Simulation Results and Analysis

#### 3.4.1. Failure Modes of Reinforced Concrete Slab

**Figure 16:**
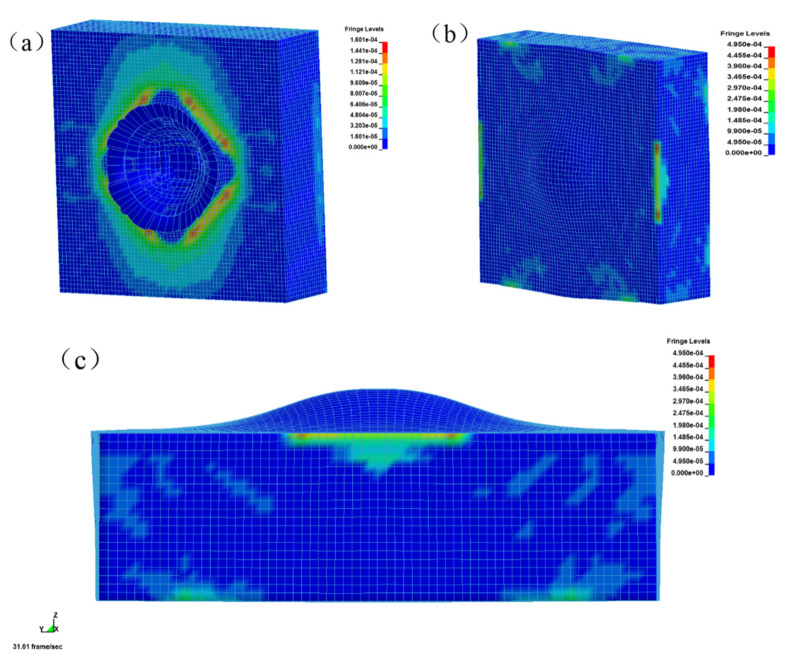
Stress nephograms of uncoated reinforced concrete slab at time t = 1000 μs: (**a**) face surface blast; (**b**) back blast surface; (**c**) side face.

The stress nephograms of the front, back, and side of the uncoated reinforced concrete plate at time t = 1000 μs are shown in Figure 16. Under the action of explosion load, the face surface blast of the uncoated reinforced concrete plate was seriously damaged by the opening pit, and the large pit appeared in the center of the explosion source, as shown in Figure 16a. The back blast surface was seriously deformed and bulged, indicating that the reinforced concrete slab was seriously damaged, as shown in Figure 16b,c. The stress wave generated by the explosion spread from the center point to the periphery. The stress reached its maximum when the explosive was detonated, and then the stress decreased gradually, but the strain remained constant. The back blast surface was subjected to the strong tensile wave transmitted by the face surface blast, and the stress was transmitted from the center to both sides. Under the action of the explosion load, the whole structure of the uncoated reinforced concrete slab had been damaged, basically losing its overall stability and bearing capacity.

**Figure 17:**
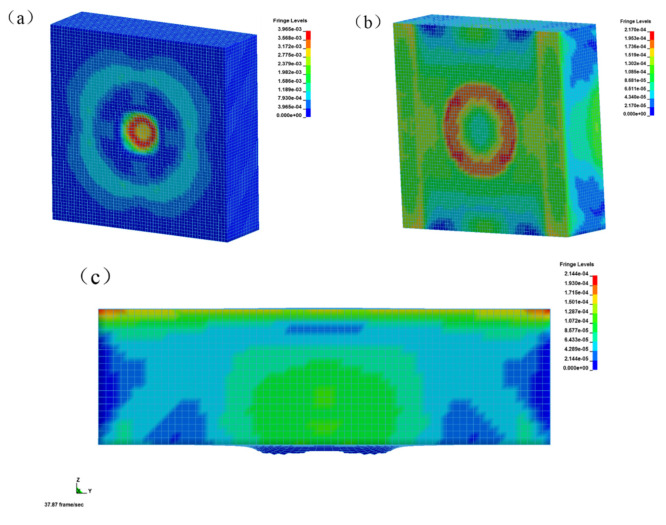
Stress nephograms of coated reinforced concrete slab at time t = 1000 μs: (**a**) face surface blast; (**b**) back blast surface; (**c**) side face.

The stress nephograms of the front, back, and side of the coated reinforced concrete plate at time t = 1000 μs are shown in Figure 17. Under the action of explosion load, there was also an explosion pit on the face surface blast of the coated reinforced concrete slab, but the damaged area was small and the depth was shallow, as shown in Figure 17a. There was no bulge on the back blast surface, indicating that the reinforced concrete slab did not produce deformation and failure, as shown in Figure 17b. The damaged area of the coating on the face surface blast was slightly uplifted, which was due to the separation of the coating and the concrete plate after the blast damage of the plate, as shown in Figure 17c. The coated reinforced concrete slab was only damaged by an opening on the face surface blast, and there was no large deformation on the back blast surface, so the overall stability was good.

**Figure 18:**
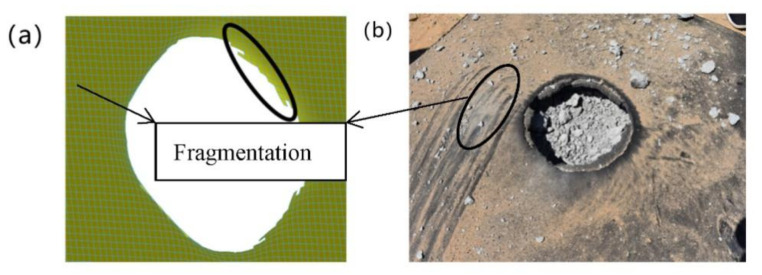
Failure mode of face surface blast polyurea coating: (**a**) simulated coating; (**b**) test coating.

**Figure 19:**
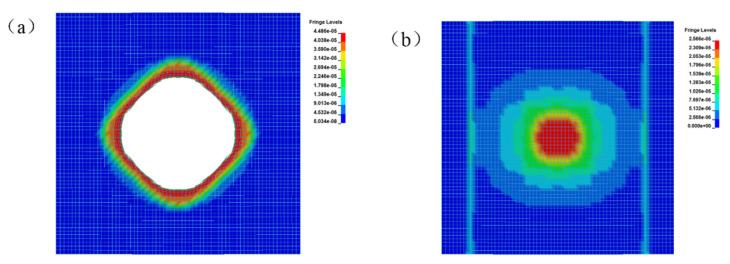
T26 polyurea coating stress nephograms: (**a**) face surface blast; (**b**) back blast surface.

In summary, a T26 polyurea coating can improve the blast mitigation ability of reinforced concrete slabs, which played a key role in the blast mitigation protection of reinforced concrete slabs. The numerical simulation and explosion test damage diagram of T26 polyurea are shown in Figure 18a,b. The central area of the coating was damaged by perforation, and the damaged area was approximately circular. The cross-section of the coating protruded upward around the extended circular area, and the destruction of the circular edge was not smooth, showing debris. The coating failure included tearing and tensile failure and mainly tearing failure, as shown in Figure 18a,b. The above thermal properties study found that the material would be carbonized when the temperature was slowly increased to 800 °C, and the carbonized layer was formed on the surface of the attached structure (Figure 18b). The carbonized layer can still be coated on the surface of the structure, but the only carbonation occurred at the perforation of the coating, and the other parts remained intact without damage, indicating that the thermal properties of the material had a certain impact on the protective effect under explosive load. The stress change in the T26 coating is shown in Figure 19. The stress in the failure area of the coating on the face surface blast was relatively large, and the coating on the back blast surface was not damaged. The stress in the central area was the largest, and was transmitted to the surrounding area in the form of a stress wave, while the stress concentration occurred at the contact between the coating and the constraint. The shock wave generated by the explosion was transmitted through the coating on the face surface blast, the reinforced concrete slab, and the coating on the back blast surface. The energy generated by the explosion was first absorbed by the deformation and failure of the front coating, then absorbed by the reinforced concrete plate, and finally dissipated by the back coating. The energy was finally completely dissipated through the attenuation of layers. In short, the T26 polyurea coating consumed part of the energy through its destruction to improve the blast mitigation ability of the reinforced concrete slab.

#### 3.4.2. Blast Mitigation Mechanism Analysis of Coating

**Figure 20:**
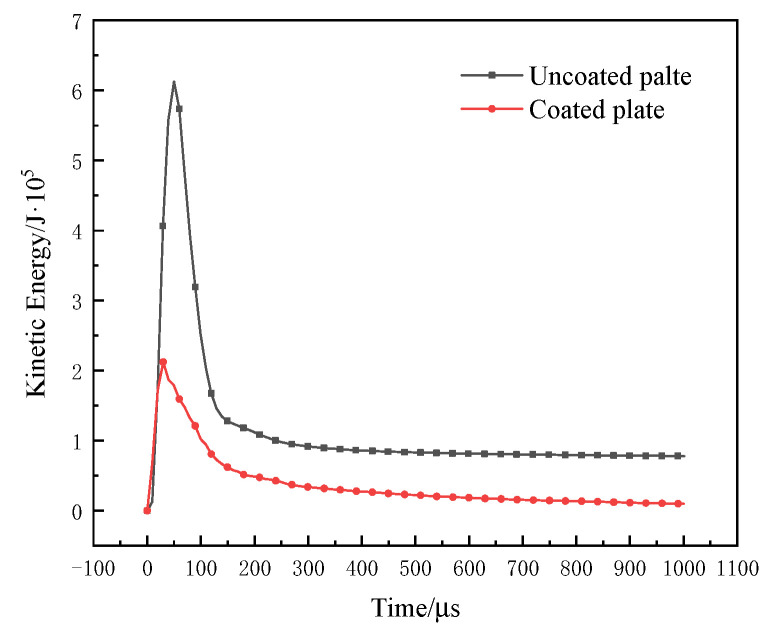
Kinetic energy curves of coated and uncoated reinforced concrete slabs.

**Figure 21:**
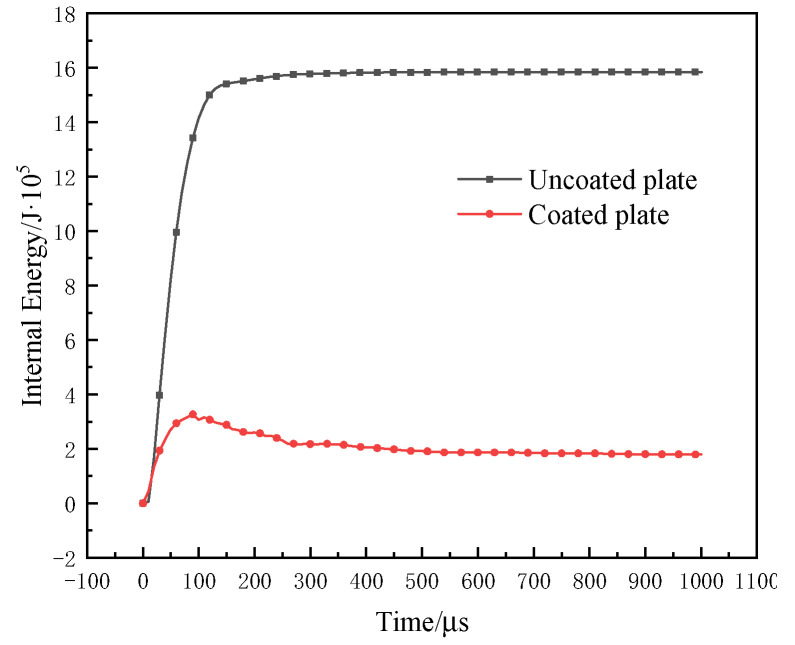
Internal energy curves of coated and uncoated reinforced concrete slabs.

An explosion can instantly produce high temperatures and strong kinetic energy that is spread around in the form of waves that penetrate reinforced concrete slabs to cause local or overall damage. When the blast wave propagates from the face surface blast of the reinforced concrete slab to the back blast surface of the reinforced concrete slab, the main failure modes of the plate are explosion pits, explosion collapse, explosion penetration, and explosion punching [23,24]. The four failure modes are mainly due to the different explosion energies, resulting in the explosion energy of reinforced concrete slabs with different intensities. The kinetic energy change curves of the coated and uncoated reinforced concrete slabs are shown in Figure 20. The kinetic energy of the uncoated reinforced concrete slab increased first and then decreased, and when the time t = 49.87 μs, the peak value was 6.13 × 10^5^ J. Additionally, the kinetic energy change trend of the coated reinforced concrete slab was similar, and when the time t = 29.96 μs, the peak value was 2.12 × 10^5^ J. Compared with the uncoated reinforced concrete slab, the peak of kinetic energy shifted and decreased by 65.42%, indicating that the presence of coating absorbed part of the energy and weakened the kinetic energy of the reinforced concrete slab, as shown in Figure 20. The internal energy variation curves of the coated and uncoated reinforced concrete slabs are shown in Figure 21. The internal energy of the uncoated reinforced concrete slab gradually increased and tended to be stable, and the maximum absorption of internal energy was 15.8 × 10^5^ J. Although the internal energy of the coated reinforced concrete slab changed little, showing a trend of increasing first and then decreasing slightly, the maximum absorption of internal energy was 3.26 × 10^5^ J. In summary, the explosion shock energy was greatly attenuated after passing through the T26 coating. The coating can reduce the kinetic energy and internal energy of the reinforced concrete slab by absorbing a large amount of the energy generated by the explosion, and ultimately improved the ability of the reinforced concrete slab to resist the explosion.

In order to further explore the energy absorption of the T26 coating, the kinetic energy, internal energy, and kinetic energy conversion rate of the face surface blast coating, back blast surface coating, and the reinforced concrete slab were shown in Table 11. The kinetic energy and internal energy of the uncoated plate were larger than those of the coated slab, and the conversion rate of kinetic energy reached 87.27%, indicating that most of the explosion energy was absorbed by the reinforced concrete plate itself, resulting in the overall collapse of the slab. For the coated slab, the kinetic energy conversion rate reached 95.36%, and the explosion energy was absorbed by the coating, which reduced the damage to the reinforced concrete slab itself. Only the face surface blast was damaged by opening the pit, and the back blast surface was intact. The T26 polyurea coating played a key role in the explosion process. For the coating on the face surface blast, in addition to the softening of the polyurea caused by the instantaneous high temperature, the sparse tensile wave of the explosive reflection wave will also damage the polyurea material, resulting in the tearing of the coating material. However, for the back blast surface coating, the polyurea coating weakened the impact tensile wave, thus protecting the concrete material from breaking and preventing explosive debris from spalling. From the above experimental results, it can be seen that the coating on the face surface blast experienced perforation damage, and it absorbed energy through its own large deformation. For the first time, the strength of the shock wave was weakened, and the propagation of energy was reduced. In addition, carbon slag, formed at high temperatures, appeared at the coating failure fracture. The carbon residue formed a carbonized layer around the circular hole and accumulated and adhered to the surface of the concrete slab, which weakened the splash of the damaged debris on the blast surface to the surrounding environment. When the explosion shock wave propagated through the concrete to the back blast surface coating, the energy decayed again. The back blast surface coating can withstand the remaining energy and can completely cover the debris generated by the concrete damage, which had a good anti-seismic collapse effect. Due to the joint action of the coating of the face surface blast and the back blast surface coating, the reinforced concrete slab could maintain good integrity and stability, which was of great significance to the blast resistance of the reinforced concrete slab.

## 4. Conclusions

(1) Compared with uncoated reinforced concrete slab, the T26-coated reinforced concrete slab had a minor damage degree and higher structural integrity under a 1.4 kg TNT explosion load, and the T26 polyurea had significant explosion protection ability.

(2) The explosion protection forms of the coating of the face surface blast and back blast surface were different. The T26 coating on the face surface blast was melted and perforated under explosion load. After melting, the coating formed a surface of carbon slag attachment, which played a role in coating and heat insulation. The T26 coating prolonged the action time and dissipation time of the explosion load by absorbing the energy of the explosion shock wave. By coating the concrete structure, the back blast surface coating restrained the explosive debris to resist the residual shock wave and reduced the secondary damage.

(3) In terms of energy conversion and dissipation, the kinetic energy and internal energy of the uncoated slab were larger than those of the coated slab under the same explosive load. The kinetic energy conversion rate of the uncoated reinforced concrete slab was 87.27%, and that of the coated reinforced concrete slab was 95.36%. The T26 coating improved the kinetic energy conversion rate of the structure and dissipated part of the kinetic energy into internal energy, thus weakening the transfer of explosive energy and improving the blast mitigation ability of the reinforced concrete slab structure.

## Figures and Tables

**Table 1:** Physical properties of t26 polyurea.

Essential Parameter	Density/g·cm^−3^	Elastic Modulus/MPa	Tensile Strength/MPa	Peel Strength/N·mm^−1^	Breaking Elongation/%
T26	0.977	84.05	25.4	75.5	451.88

**Table 2:** Explosion test conditions.

Test Group	Coating	Coating Thickness/mm	Coating Method	Blasting Distance/mm	Restraint Mode
1	-----	0	-----	0	Pin constraint
2	T26	10	Full spraying	0	Pin constraint
3	T26	10	Full spraying	50	Pin constraint

**Table 3:** Comparison analysis method of macroscopic morphology.

Test Conditions	Coating	Coating Thickness/mm	Failure Plane	Macroscopic Morphology Comparison
1	——	0	Face surface blast	Failure mode and diameter
			Back blast face	Failure mode and diameter
2	T26	10	Face surface blast	Failure mode and diameter
			Back blast face	Failure mode and diameter

**Table 4:** MAT_PIECEWISE_LINEAR_PLASTICITY CARDS.

MID	RO	E	PR	SIGY	ETAN	FAIL	TDEL
3	0.977	0.00084	0.4	0.0014	0.00025	1.4	0
C	P	LCSS					
40	5	10001					

**Table 5:** Johnson–Holmquist–Cook concrete CARDS.

MID	RO	G	A	B	C	N	FC
1	2.44	0.1486	0.79	1.6	0.007	0.61	0.00048
T	EPS0	EFMIN	SFMAX	PC	UC	PL	UL
0.00004	1.0	0.01	7.0	0.00016	0.001	0.008	0.10
D1	D2	K1	K2	K3	FS		
0.04	1.0	0.85	−1.71	2.08	0.8		

**Table 6:** MAT_PLASTIC_KINEMATIC CARD.

MID	RO	E	PR	$\sigma_{0}$	E_P_	F_S_
2	7.8	2.1	0.3	0.00235	0.021	0.8

**Table 7:** MAT_NULL CARD.

MID	RO	PC	MU	TEROD	CEROD
5	0.000129	0	0	0	0

**Table 8:** EOS_LINEAR_POLYNOMIAL CARD.

MID	C0	C1	C2	C3	C4	C5	C6	E0	V0
5	−1 × 10^6^	0	0	0	0.4	0.4	0	2.5 × 10^−^^6^	1

**Table 9:** MAT_HIGH_EXPLOSIVE_BURN CARD.

MID	RO	D	PCJ
4	1.63	0.8425	0.2995

**Table 10:** EOS_JWL CARD.

MID	A	B	R1	R2	OMEGA	E0	V0
4	5.817	0.06815	4.10	1.0	0.35	0.09	1.0

**Table 11:** Energy changes of reinforced concrete slab and coating.

Parameter	Uncoated Slab	Coated Slab	Surface Facing Blast Coating	Back Blast Surface Coating
Maximum kinetic energy/J	6.13 × 10^5^	2.12 × 10^5^	3.39 × 10^4^	188.71
Energy deposit/J	7.8 × 10^4^	9.83 × 10^3^	1.79 × 10^2^	14.10
Absorption of internal energy/J	15.8 × 10^5^	3.26 × 10^5^	182.79	58.60
Kinetic energy conversion rate/%	87.27	95.36	99.47	92.52

## Data Availability

The data that support the findings of this study are available from the corresponding author upon reasonable request. Informed consent was obtained from all subjects involved in the study.
